# Sciatica

**Published:** 1888-05

**Authors:** 


SCIATICA.

This patient is 45 years of age. She complains of pain in the left side. This pain she says begins in the small of the back and shoots down the left leg. When I press over the trochanter she complains of pain; when I press into the popliteal space she complains of pain; also when pressure is made over the head of the fibula. She complains also of pain on the inner side of the foot, over the internal malleolus. We will see if there is any difference in the size of the limbs. We will select two corresponding points on the right and the left legs. The left leg measures two inches less in circumference than the right. The pain is always worse at night. Notice the difference in the quality of this pain, and the location of it, as compared with the cases of locomotor ataxia that appeared before you a week ago. This pain is distributed more or less over the whole leg, following the course of the nerve-trunks; it is not localized. It has been followed by atrophy of the limb.

This is a case of sciatica. We will try to find out what the cause of it is. She is the mother of thirteen children, eight of whom are living, and has had five miscarriages. We elicit no history of rheumatism, and no history of syphilis. Her menstrual periods have been less profuse, and at longer intervals than formerly; she is approaching the menopause. Her urine was found on examination to be abnormally acid and loaded with uric acid and urates. The condition of the menses and urine furnishes a clue to the cause and suggests the line of treatment. The menstrual period is the period of purification; there is a large amount of waste material eliminated at this time, and when the function ceases there is more or less disturbance produced in the nervous system by the circulation in the blood of this excrementitious matter, and neuralgia is a common result of this retention. We will therefore prescribe for this patient mild alteratives, laxatives, and tonics, to promote the removal and arrest the formation of these morbific products.

R:

Sodii salicylatis, drachms..........5
Sodii iodidi " .........2½
Syrupi rhei et potassii comp., oz...1
Aquæ Gaultheriæ, oz..............4

M. Sig.--One teaspoonful before each meal, in water.

R:

Quiniæ bromidi, oz..............1½
Acidi arseniosi, gr..............1
Strychniæ sulphatis, gr... .......1
Ext. aconitæ, gr................15

M. Ft. in capsulæ No. XXX.

Sig.—One after each meal.

The limb should be enveloped in cotton batting covered with oiled muslin or rubber cloth, first rubbing it with the following anodyne liniment:

R: Chloral hydrate.

Camphoræ.

Ol. cajuputi ana, one ounce.

M. Sig.—Use as a liniment.

Massage is an admirable adjunct to the treatment of sciatica.

The manner of giving massage in cases of this kind is to take the limb in both hands, press one's fingers deeply into the limb, if possible, against the sciatic nerve, and then by friction along the course of the nerve through the thigh and down to the calf of the leg, grasping the limb and pressing the fingers deep into the tissues. A great many cases of sciatica are due to a congested condition of the nerve, and that may be the result in this case. There are cases again in which there is inflammation of the nerve. You know the nerves are liberally supplied with blood vessels, and congestion of these blood vessels, at least, accounts for many cases of neuralgia. The use of the constant galvanic current, putting the negative pole over the spine and the positive over the tender points, permitting the current to pass four or five minutes through each painful point, greatly relieves such patients. Where the pain is violent, hypodermic injections of morphine may be used in some instances, injecting the morphine as close as possible into the nerve-trunk. Opium will relieve some of these cases, but it is a bad practice to habitually use morphine or any preparation of opium for the relief of neuralgias or sciatica. It leads a great many of these people into morphine habits. Having had an attack of neuralgia, they are liable to another, and the use of morphine in nine cases out of ten will create the injurious habit—a habit worse than the effects of the disease.

579 West Jackson Street.

